# Teeth Rehabilitation and Nutritional Influence on Diabetic Patients: A Review

**DOI:** 10.7759/cureus.46182

**Published:** 2023-09-29

**Authors:** Dalea M Bukhary

**Affiliations:** 1 Department of Oral and Maxillofacial Prosthodontics, King Abdulaziz University, Jeddah, SAU

**Keywords:** tooth loss, denture, prosthodontics, nutrition, mastication, occlusion, diabetes

## Abstract

Diabetes mellitus (DM) is a globally prevalent endocrine and metabolic disorder characterized by hyperglycemia. Its complications significantly impact both the quality and longevity of the patient’s life with a substantial burden on the healthcare system. Missing teeth make individuals more susceptible to malnutrition compared to those with functional teeth. This is especially true for people with diabetes, as the condition is closely linked to both oral health and food intake. Natural teeth loss can significantly reduce an individual's ability to chew food, leading to a decrease in the quality and quantity of their nutrition. Prosthodontics is a dental specialty that replaces missing teeth with artificial ones. Replacing missing teeth may prevent the risk of malnutrition due to decreased ability to chew. Good oral health is important for overall health, especially for those with conditions such as diabetes. Artificial teeth replacement may improve nutrition intake by improving chewing ability. Therefore, the ultimate objective of rehabilitating a patient's oral cavity is to replace the shape and function in terms of chewing performance to a degree that is as close to normal as feasible. The purpose of this review is to explore the literature showing the link between natural teeth loss and nutrition in individuals with DM, with a special focus on prosthodontic management. Several oral complications occur in diabetic patients specifically teeth loss, which in turn affects mastication function. This in turn cause malnutrition and affect glucose level. It is imperative for healthcare providers to take an interdisciplinary approach in order to improve the dental and nutritional status and overall well-being of DM patients.

## Introduction and background

Diabetes mellitus (DM) is a globally prevalent endocrine and metabolic disorder. It is characterized by hyperglycemia resulting from defects in insulin either in its secretion, action, or both [1]. The complications of DM include damage to the vasculature and microvascular (retinopathy, nephropathy, and neuropathy) and macrovascular (cardiovascular diseases, such as coronary artery, cerebrovascular, and peripheral artery diseases) diseases. These complications significantly impact both the quality and longevity of the patient’s life and mortality rate [2]. In addition, they place a huge burden on the healthcare system [3]. The prevalence of DM is continuously increasing in modern society. According to a national survey in Saudi Arabia, the estimated prevalence rates of DM were 14.8% for males and 11.7% for females in 2013. It increased with age and ranged from 7.8% among those aged 25-34 to 50.4% among those aged 65 and older [4]. There are two main types of DM: type 1 (DM1) and 2 (DM2). Diabetes (DM1), has an absolute deficiency of insulin secretion and loss of insulin-producing β-cells in the pancreas by responding to a multifactorial pathogenesis linked to an autoimmune aggression mediated by autoantibodies. It commonly occurs early in life and accounts for only 5-10% of the population. Meanwhile, DM2 is more prevalent, especially in adults, and accounts for ~90-95% of the population. It is caused by a combination of resistance to insulin action and an inadequate compensatory insulin secretory response. An important note for this type is at a specific level of hyperglycemia, and without clinical symptoms, pathologic and functional changes occur in several tissues in the body without detecting DM [1,5]. Symptoms of marked hyperglycemia are typically characterized by the "3 Ps" triad, namely, polyphagia, polydipsia, and polyuria [6,7].

DM complications are divided into short-term (acute) and long-term (chronic) complications; oral complications fall into the chronic long-term complications [2]. Furthermore, several other pathogenic mechanisms, such as insulin resistance, dyslipidemia, hypertension, and immune dysfunction, can contribute to DM complications, particularly those in the oral cavity [2]. Analysis of glycated hemoglobin (HbA1c) in the blood provides evidence about an individual’s average blood glucose levels during the previous two to three months, which is the predicted half-life of red blood cells [8]. The HbA1c is recommended as a standard of care (SOC) for testing and monitoring diabetes, specifically DM2. In addition, HbA1c correlates and predicts the risk of long-term diabetes complications [9,10].

This review focuses primarily on understanding the factors affecting diabetes patient nutrition in relation to remaining teeth and prosthodontics treatment. Oral health is important as it reflects on the general health of the person. Several oral complications occur in diabetic patients specifically teeth loss, which in turn affects chewing, and mastication function. This in turn causes malnutrition and affect glucose level.

## Review

A thorough search of electronic databases was conducted, including Science Direct, PubMed, Scopus, and Google Scholar, on articles published in the last 15 years. Our search was limited to studies within keywords such as diabetes, occlusion, mastication, nutrition, prosthodontics, denture, and tooth loss, and assessing the quality of the studies was performed.

Patients with DM are susceptible to a wide range of oral complications, such as periodontal disease, dental caries, xerostomia, hyposalivation, dry mouth, oral lesions (related to candida or unrelated to candida), oral cancer, taste alteration, halitosis, apical periodontitis, and peri-implant disease [11]. The retention of functional dentition (FD) comprising natural teeth is an indicator of good health and nutrition intake. Patients with DM are more likely to have untreated dental diseases and periodontitis, which can lead to tooth loss [12,13]. As mentioned in the previous section, the relationship between DM and oral health problems, such as the loss of natural teeth, results in compromised dentition [14]. The most common causes of teeth loss are periodontitis and dental caries. Periodontitis is microbially-associated, host-mediated inflammation and its risk is increased by several factors that cause alterations in the immune function, thereby inducing periodontitis leading to loss of periodontal ligaments attachment around the teeth root, bone loss, and consequently loss of a tooth [15]. Dental caries is caused by the bacteria of dental plaque that produces acid on the tooth surface and destroys the tooth tissue (enamel and dentin), resulting in tooth caries (or decay). In addition, diabetic patients may have more cavities due to lower saliva flow and higher glucose levels in the parotid saliva [15].

DM and teeth

According to the literature, number of retained teeth was linked to the development of DM. individuals tend to have a decreased risk of developing DM when the number of retained teeth increases [16,17]. In a longitudinal study done in 2020, Chang et al. found that among Korean adults, having more than 15 missing teeth was positively correlated with developing new-onset diabetes [16]. This is in line with a finding by Abe et al. that people seem to have a lower risk of acquiring diabetes the more teeth they still have [17]. When all teeth are absent, this is called edentulism. As a consequence of teeth loss, the bone (which is now termed residual ridge ) starts to resorb continuously. Bone loss occurs following tooth extraction and continues over time, with the mandibular residual ridge being particularly susceptible to resorption than the maxillary arch. The resorption in the mandible occurs at a rate about four times that of the maxillary residual ridge. Therefore, the primary intraoral complication of edentulism is chronic residual ridge resorption (RRR), which can lead to changes in esthetics and soft tissue profile [18], leading to protrusion of the chin [19,20]. Studies have reported the relationship between edentulism and developing DM specifically in DM 2 [21]. A Mexican study investigating the relationship between complete edentulism and DM demonstrated that edentulous patients have a 1.82 times greater risk of developing diabetes than dentate individuals [22]. Additionally, a cross-sectional study revealed that older men without teeth had a four times greater risk of developing non-insulin-dependent DM than those with partial or complete dentitions, regardless of age or race [23]. Based on research, it was indicated that there were various factors related to oral health that may affect masticatory performance. However, the number of natural/remaining teeth had the most significant positive impact on masticatory performance. However, it should be noted that evidence supporting this conclusion is of very low quality [24].

Remaining teeth and nutrition intake

Maintaining good oral health is crucial for ensuring proper nutrient intake [25]. The oral health status may also be an essential factor influencing the diet in terms of food type, choice, and eating behavior. Tooth loss is associated with poor consumption of foods, such as meat, nuts, dairy products, fruits, and vegetables, and suboptimal intake of a range of micronutrients [26]. Malnutrition especially in older individuals may result in the avoidance of foods that are harder, stringier, and more difficult to chew, thereby resulting in the preference for softer food and nutritionally poor food choices.

The literature produced conflicting results about the link between a person's nutrient intake, food preferences, and the quantity of remaining natural teeth. These studies examined a variety of aspects such as nutritional status assessment, dietary evaluation and dietary diversity score computation, dentition state assessment, and food. The number of remaining natural teeth has been shown to influence nutrition intake by reducing the intake of dietary fiber, protein, and basic nutrients from fruits and vegetables [27,28]. Older adults with 0-9 remaining teeth had a lower intake of total and animal protein compared to those with ≥20 remaining teeth. However, eating plant proteins was not associated with fewer remaining teeth and dental prosthesis use [29]. The relationship between nutrition and the presence of teeth in specific FD has been investigated in the literature. A systematic review and meta-analysis looked at adults aged ≥60 years old living in developed countries and studies using validated tools to assess the risk of malnutrition in older adults. They found that older adults with tooth loss are at greater risk of malnutrition than those with functionally adequate dentition. The authors suggest that, compared to older persons who were dentulous or had functionally adequate dentition, those who were fully edentulous or lacked functional teeth had a 21% higher risk of malnutrition or being malnourished [30]. Additionally, several studies in different countries (China, Japan, and Northern Ireland ) have reported the presence of a relationship between the number of teeth and dietary diversity. The studies agreed that the presence of less than 20 natural teeth affects diet and the diversity of food selection. In addition, the studies emphasized the importance of denture use and showed improvement in diet by increasing the consumption frequency compared to non-wearers. The diversity in food is essential to prevent physical frailty and malnutrition by preventing impairment in skeletal muscle formation and muscle function maintenance [31,32].

Remaining teeth and mastication

Mastication is a process by which food is crushed and ground and requires complex biomechanical coordination of the lips, cheeks, tongue, palate, teeth, saliva, temporomandibular joint, and masticatory muscles [33]. Maintaining masticatory function might play a crucial role in general health and reduced or the lack of masticatory efficiency is considered a major issue among adults. It has been associated with insufficient nutritional intake [24]. Individuals unable to fully masticate due to teeth loss or ill-fitting dentures have insufficient dietary intake due to lower chewing efficiency including lower fiber, protein, and minerals. Chewing difficulty affects the type of food, such as dietary fibers and proteins requiring greater masticatory forces for ingestion. Therefore, lowering chewing efficiency affects the selection of foods in individuals with tooth loss [34]. Studies showed that adults with teeth loss or completely lost their teeth "edentulous" may avoid or stop eating foods that they find difficult to eat such as raw vegetables, apples, and nuts [26]. Additionally, the decrease in the intake of food that is tough to chew may result in the swallowing of food without proper chewing, thereby causing malabsorption. This can further result in malnutrition and increased reliance on softer foods, generally carbohydrates and saturated fats, which may increase the risk of occurrence and progression of diabetes [30,31,35,36]. Studies have also shown that there is an association between mastication and the prevalence of diabetes specifically DM2 and the progression of its complications [37].

Previous reports suggested that at least 21 or more teeth are required for adequate chewing ability [38]. The mechanism behind it is that the loss of teeth affects both the sensory and motor components of the masticatory process. The orofacial and central nerve systems are both involved in the process of mastication. In order for the masticatory muscles to contract and coordinate as one unit, the periodontal mechanoreceptors are neural receptors that help in this process. Therefore, following tooth loss, impairment in masticatory performance is related to the lack of sensorial feedback and muscular atrophy [39].

Mastication and DM

A cross-sectional study conducted in 2022 by Hamamoto et al. found a significant correlation between masticatory efficiency, remaining teeth, and diabetic neuropathy in a cross-sectional investigation. This showed that DM2 patients who developed diabetic neuropathy had significantly lower masticatory efficiency. The masticatory effectiveness was quantified using a gummy jelly and the Gluco Sensor GS-II (GC Corporation, Tokyo, Japan), and the quantity of eluted glucose in the filtrate was calculated and used as a measure of the average masticatory efficiency, which was 172.4 ± 60.0 mg/dL. They evaluated the status of diabetes and periodontitis and discovered a positive correlation between masticatory efficiency and the number of teeth remaining [40]. Interestingly, the authors related the masticatory dysfunction in DM patients results to two possibilities: 1) sarcopenia as DM2 is associated with sarcopenia, whereby sarcopenia may cause masticatory muscle mass reduction and induces masseter muscle atrophy and chewing dysfunction, and 2) impairment for mastication could be impaired trigeminal nerve function due to diabetic neuropathy (as muscles for mastication, consisting of the masseter, temporal, medial pterygoid, and lateral pterygoid muscles, are innervated by the trigeminal nerve). Therefore, when these mandibular motor fibers are affected, the patients may complain of weakness in chewing [40].

Various studies have discovered a correlation between the number of teeth remaining (NT) and masticatory function (MF) with DM status [17,35]. In 2021, Abe et al. conducted a cross-sectional analysis of elderly Japanese adults. The goal was to investigate the impact of both the NT and MF as oral health indicators on the development of DM in older adults living in the community. Dental hygienists evaluated both NT and MF. The measurement is based on the number of gummy jelly pieces gathered after chewing. DM status was determined by a hemoglobin A1c level of 6.5% or self-reported diabetes. The study found that the NT and a higher level of MF were negatively associated with DM. The NT was associated with DM status, while MF was also associated with DM status. The study measured MF based on a combination of factors, including the number of remaining natural teeth and the use of dentures. Although the authors did not compare with and without denture patients, they suggest that the use of dentures may lower the risk of losing the remaining teeth and improve MF. Therefore, it was not examined in their study the associations of MF with dentures. The study suggests that MF and NT were significantly associated with DM, based on either HbA1c 6.5% or self-reported DM [17]. Additionally, Yamazaki et al. discovered that Japanese men with high masticatory performance had lower odds of developing diabetes by using color-changing chewing gum. The study suggests that eating slowly and preserving high masticatory performance by preventing tooth loss or maintaining dental prostheses might prevent the occurrence of diabetes [35]. Literature showed that, besides the effect of remaining teeth number on nutrition, the occlusal condition (occlusion) and occlusal surfaces of the teeth play an important role in enabling food fragmentation and bolus formation [41]. Therefore, maintaining occluding surfaces enhances the masticatory performance [42].

Kosaka et al. conducted a study that pointed out a significant connection between the masticatory function of elderly Japanese individuals and their number of functional teeth and occlusal support areas. To assess the occlusal function of patients with incomplete or missing teeth, the authors employed the Eichner index to categorize the occlusal support and identify the functional occlusal support areas [43]. Additionally, Tanaka et al. showed that masticatory performance in elderly adults is influenced by occlusal state (i.e., the number of occlusal supports and the presence or absence of wearing removable dentures). They examined glucose extraction when older persons chewed gummy jelly by using their usual chewing side. They showed that wearing a removable denture may improve and regain the masticatory performance resembling natural teeth [42]. A 12-month longitudinal study in a sample of Thai older adults reported a relationship between FD, which is defined as containing ≥10 occlusal contacts, and changes in dietary patterns (DP) in older adults; however, the results were not significant. They used a 154-item semi-quantitative food frequency questionnaire (FFQ) to assess the intake of food and beverages in the last month. FD was defined as having all six anterior functional tooth units (FTU), plus at least four (out of a maximum of 16) posterior FTU [44].

According to a 2015 study by Inomata et al., after adjusting for possible confounding factors, they reported that individuals who wore removable partial dentures (RPDs) with at least 20 functional occlusal units consumed a greater amount of beneficial nutrients, including vegetables, n-3 fatty acids, calcium, vitamin A, and dietary fiber, compared to those who did not wear RPDs [45].

Remaining teeth and glycemic level control

As mentioned in oral complications of DM, studies have revealed a correlation between poor oral health and hyperglycemia, indicating that an unfavorable glycemic profile may increase the risk of periodontitis and tooth loss [46]. Several studies have reported relationships between teeth loss, mastication performance, and glycemic control. Longitudinal research revealed that the risk of caries development increases as the HbA1c level increases and that the rate of missing tooth surface accelerated in people with DM than in people without DM, even when they attained HbA1c 7.0% [47]. As the loss of teeth changes the food type, studies showed that the selection of foods shifts toward soft sugar-rich meals. In addition, there is a decrease in the chewing time of the meal, leading to insufficient secretion of insulin and an increase in blood glucose levels [48]. Additionally, Iwasaki et al. demonstrated a link between unhealthy eating habits and a poor glycemic profile. They looked into the connection between eating habits (such as skipping breakfast and eating hastily) and poor glycemic control in Japanese adults aged 40 or older with hemoglobin A1c (HbA1c) levels below 6.5% who took part in a health questionnaire. After follow-up, they characterized poor glycemic control as HbA1c of 6.5%, increases in HbA1c of 0.5%, and/or taking diabetes medication. The risk of having poor glycemic control was higher in males [25]. According to a study by Fujishiro et al., there may be a link between self-reported chewing issues and HbA1c levels below 7.0% in senior Japanese community dwellers. Using a self-reported chewing problem implemented in a health checkup, Fujishiro et al. conducted a cross-sectional study on adults 65 years of age who had undergone an annual health checkup. According to the study, chewing issues affected 10.4% of the aged population (106 out of 1,018 participants) undergoing an annual health checkup. The HbA1c values of people with chewing issues were statistically significantly higher than those of people without such issues. Additionally, HbA1c 7.0% has been shown to greatly increase the risk of self-reported chewing issues even in healthy individuals. Additionally, they discovered strong links between eating quickly, and smoking status. The study was limited, nevertheless, because information pertinent to the usage of dentures and the history of dental care was not available because these factors were not part of the system for yearly health examinations. This study was done in an urbanized area, and objective evaluations of oral health were not performed in this study [49]. An interesting cross-sectional study performed on a Chinese population investigated the relation between the number of functional occlusal areas( classified by the Eichner index) and the prevalence of DM2. The study suggested that, as the occlusal support decreases, this is related to an increase in the blood glucose concentration and consequently the prevalence of DM2 [41].

Prosthodontics management considerations

As shown in the above sections, natural teeth loss and occlusal status affect mastication and nutrition status, especially for DM individuals. Therefore, oral health workers specifically prosthodontists may consider this in managing DM patients. Oral rehabilitation using oral prosthesis includes fixed crowns, partial dentures, or complete dentures (CDs) either supported by natural teeth or dental implants. In this review, the focus will be on removable prosthodontics management literature and its effect on patient nutrition. There are conflicting data regarding the functional benefits of prostheses used to replace missing teeth. Several studies have reported that dental prostheses did not improve nutrition [50,51].

In 2001, Hamada et al. conducted a study to compare the diets of diabetic patients without teeth before and after receiving new dentures. The patients were given either traditional dentures or implant-supported overdentures. The study included patients with good metabolic control, with or without insulin. The researchers recorded the patients' dietary intake for a week before and six months after treatment completion. They analyzed the average daily intake of 28 essential nutrients and compared their intake with the recommended daily allowance. The study found that new dentures, whether traditional or implant-supported, did not significantly alter the dietary intake of patients with good metabolic control of diabetes, regardless of insulin use. These findings align with previous studies that suggest the clinical quality of dentures or other prosthetic treatments has no effect on dietary intake [52]. Moreover, Yamazaki et al. reported that, in a narrative appraisal, a meta-analysis focusing on comparing conventional dentures with the overdenture treatment provided for older patients did not show changes in BMI, albumin, or vitamin B12 when followed up to six months after treatment. The study suggests that the result could be explained by the multifactor effect that may influence the result of the comparison. In the older population, nutritional inadequacy could be influenced by physiological, pathological, sociological, and psychological factors [50].

On the contrary, several studies support the positive effect of removable prostheses on nutrition intake. Kusama et al.'s 2021 study demonstrated that dental prostheses have a protective effect against malnutrition attributed to tooth loss by reducing the risk of weight loss in community-dwelling older adults in Japan [53]. Other studies performed on the Japanese population reported that older adults show a decreased risk of malnutrition when provided with partial dentures. Studies reported that using dentures may improve masticatory performance and nutritional intake quantity and quality, thereby affecting their dietary selections. The denture wearers' nutrient intake in comparison to dentate patients had dietary fiber, fruits, and vegetables [54,55].

Additionally, a cross-sectional study by Kusama et al. demonstrated that prosthodontic treatment contributed to the maintenance of protein intake in older adults with severe tooth loss. The participants completed a self-reported questionnaire. They used % energy intake (%E) of the total protein as the outcome and the use of dental prostheses and the number of remaining teeth as explanatory variables. Compared with intact natural teeth, individuals with partial or complete prostheses consume fewer fruits and vegetables, have a higher risk of developing malnutrition, and are less likely to meet the recommended nutrient intake levels, thereby demonstrating low vitamin A, riboflavin, zinc, and folate intake levels [26]. Concerning removable denture quality studies, a study looked at the relationship between denture quality and dietary intake of nutrients. A study in Australia found no association between denture quality and inadequate dietary intake of nutrients [26]. Moreover, Shinkai et al. measured as good or bad by looking at the denture quality in terms of retention, stability, and tooth wear of the posterior artificial teeth. The study found that the energy, protein, vitamins, folate, iron, and fiber intake were not significantly different if the denture was categorized as good or bad [56]. Interestingly, the replacement of teeth in a single arch showed a negative effect on MF. A cross-sectional study on 179 nursing home residents with a mean age of 78.9 years demonstrated that CD in only one jaw nonfunctional dental prosthesis in edentulous participants negatively affected the MF. The stability, retention, occlusion, vertical dimensions, and defects were considered during the functional assessment of the dental prostheses. MF data were determined by assessing the (a) masticatory performance using two-colored chewing gum and (b) swallowing thresholds by counting the number of peanut chewing cycles [22]. Compared to complete dentate individuals, the maximum biting force of CD wearers is 1/7th to 1/4th, and the chewing ability with CD is approximately 20% of the force of an average person. Therefore, using CD reduces chewing ability and affects the quantity of foods such as vegetables. These changes in food consumption as mentioned above may cause nutritional deficiencies and affect glycemic control [11,52]. Studies show that patients could chew properly with new dentures within two months, as a new memory pattern is created in the oral muscles [57]. Bousiou et al. demonstrated that poor masticatory performance in community-dwelling persons aged > 60 years was associated with fewer teeth, the use of CDs, and an increased prevalence of severe tooth mobility [58]. Despite no evidence of a clear pattern of the impact of wearing dentures on the measured dietary intake in individuals with tooth loss, wearing dentures can have a positive impact on the nutritional status and pleasure of eating [59]. Although oral rehabilitation of edentulous patients with CDs reportedly improves their masticatory performance. The number of chewing strokes required to break down test food was approximately four times greater for denture wearers compared to individuals with natural dentitions [57]. Sahyoun et al. reported that self-perceived patients with poor CD quality had significantly lower healthy eating index (HEI) scores (i.e., lower intake of fruits and vegetables, lower dietary quality scores, and less variety in their diet) than those with five or more pairs of posterior teeth [60]. The HEI tool evaluates the overall quality of one's diet by categorizing them as poor or good diet, a score of 51-80 is considered to need improvement, and anything above 80 is considered good. A study analyzed the nutritional status of patients with teeth (opposing pairs of posterior teeth) and those wearing dentures. According to the findings, patients who had less than four pairs of opposing posterior teeth were more likely to experience malnutrition. Surprisingly, individuals with CD scored better compared to those who had no replacement teeth in the posterior region or those with one to four opposing pairs of posterior teeth. Nevertheless, patients without teeth were still at risk of inadequate nutrition [61].

A study demonstrated that the US adult population using dentures may be at a nutritional disadvantage compared to completely dentate patients, even after adjusting for potential social and behavioral factors (such as smoking status and vitamin and mineral supplement use). This study used an FFQ, 24-hour quantitative dietary recall, and biochemical analyte levels to determine the intake of nutrient-laden vegetables that are also high in dietary fiber. They demonstrated that the intake of certain nutrient-rich foods and serum beta-carotene, folate, and vitamin C levels were significantly lower in denture wearers [62].

RPDs should be used in specific partially dentate diabetic situations, rather than fixed dental prostheses or implant-supported prostheses to replace lost teeth. This is due to the difficulty of implant osseointegration in patients with poorly managed diabetes [63] or periodontally damaged abutment teeth [64]. In addition, DM2 is more prevalent in lower socioeconomic groups, and treating partially dentate cases with fixed dental prostheses, such as implant-supported prostheses, may be challenging in individuals with a lower socioeconomic status. Therefore, patients with diabetes are more likely to receive an RPD than an implant-supported fixed dental prosthesis [65]. A 2019 study conducted by Lee et al. discovered that Korean adults who were 50 years or older and wore CDs were more likely to have uncontrolled diabetes than those who used other types of dental prostheses. Their research aimed to explore the effects of removable dental prostheses on diabetes and glycemic control in the Korean population. Patients were categorized into three groups: Group NF, which did not use removable dental prostheses; Group RPD, which used RPD but not CD; and Group CD, which used at least one CD. Diabetic patients were identified based on fasting plasma glucose levels of at least 126 mg/dL, taking insulin or anti-diabetic medication, being diagnosed with DM by a physician, and having an HbA1c level of 6.5% as a cut-off point for uncontrolled diabetes. The study observed that the use of removable dental prostheses had a significant association with diabetes and glycemic control in men. Men who wore CD (Group CD) had a 1.49-fold greater risk of having diabetes compared to those in Group NF [11].

Recommendations

Teeth rehabilitation for DM patients has been shown to have an impact on their nutrition and, therefore, their whole health. An interdisciplinary approach involving DM care may improve oral health, nutrition, and the quality of life for diabetic patients. Therefore, the following recommendations were collected from the literature to improve and prevent the nutritional deterioration of DM patients and provide successful treatment outcomes:

(1) When examining diabetic patients with natural teeth loss or dentures, healthcare providers, such as endocrinologists and dentists, should consider the potential risk of uncontrolled diabetes; therefore, food intake should be assisted. Lee et al. demonstrated a high prevalence of uncontrolled diabetes in patients with RPDs compared to those with other types of dental prostheses, especially in Korean men and adults above the age of 50 years [11]. Therefore, the Nutrition and Dietetics Guideline suggests maintaining nutrition intake and preventing malnutrition, especially in DM patients [66]. Furthermore, elderly edentulous individuals often experience changes such as decreased muscle strength, which can lead to nutritional deficiencies. Thus, a higher incidence of gastrointestinal disorders and malnutrition has been reported in this age group [57].

(2) Inadequate eating habits may be investigated, whereby studies showed the link between malnutrition, mastication, and DM. Adequate eating habits may reduce the incidence of diabetes by improving glucose metabolism after meals [35]. Malnutrition may lead to low levels of nutritional biomarkers, such as serum albumin. Albumin is a predictive indicator of frailty and lower survival [29]. A positive association between fasting plasma glucose levels and missing natural teeth in elderly rural people was found by Lee et al. Thorough mastication elicits a lower postprandial plasma glucose concentration because of the potentiation of early-phase insulin secretion. Slow eating leads to lower postprandial concentrations of specific peptides such as anorexigenic gut peptide YY and glucagon-like peptide 1. However, few studies have demonstrated a direct relationship between mastication and glucose metabolism. Physicians should check for oral conditions and chewing problems in DM patients with HbA1c ≥7.0% [49].

(3) Regarding nutrition counseling, dental healthcare providers should consider collaborating with dietitians to provide nutritional care, and dietitians should consider oral health as a risk factor when assessing nutrition in older adults [26].

(4) The type of prosthesis may also have an impact on the type and frequency of food recall, especially if an RPD is used [11]. Studies showed that the type of food in individuals with prostheses has a higher total and saturated fat intake with a higher percentage of energy levels from fat compared to those with natural teeth. Lee et al. demonstrated that edentulous older adults had significantly higher fat and saturated fat levels and percentage of energy levels from fat than dentate adults [67]. However, Hamada et al. demonstrated that the type of prosthesis does not affect nutrition intake or dietary habits in DM patients with acceptable metabolic control, regardless of whether metabolic control was maintained with or without insulin. These habits are likely to develop gradually and can be influenced by many socioeconomic, cultural, and behavioral factors. Therefore, repeated nutritional counseling should be provided for DM patients [52].

(5) Although both genders are treated and managed equally, studies suggest a focus on diabetic females. Studies showed that gender affects masticatory performance when teeth are lost. Studies have shown that women who wear dentures tend to experience a more significant decrease in their chewing ability than men. This is thought to be attributed to men's naturally stronger jaw muscles, which play a vital role in breaking down food into smaller particles, especially for those who are new to denture wear. Furthermore, osteoporotic postmenopausal women may have lower bone quality, causing reduced height in mandibular bones. If the alveolar ridges are severely resorbed, it can negatively impact denture outcome by affecting retention, chewing ability, and overall satisfaction with treatment [57]. For females in particular, reduced occlusal force and decreased swallowing function were significantly associated with the presence of frailty [68].

(6) Prevention and management of diabetic complications have become a major aspect of modern diabetes care. Proper chewing is crucial for successful diabetes treatment, especially for those with diabetic neuropathy. Difficulty chewing may hinder even with a proper dietary plan. Dentists and nutritionists should consider a patient's chewing ability when recommending diet therapy to control high blood sugar levels [40]. Medical nutrition therapy is now an essential aspect of adult diabetes treatment. The recent ADA recommendations for patients with DM2 require a reduction of 1,050-2,100 kJ from the RDA levels with caloric contributions of less than 30% from total fats, between 10% and 20% from proteins, and 60%-70% from carbohydrates and monounsaturated fats. Therefore, it is important for dentists to repeatedly provide nutritional counseling to their patients [52].

(7) Healthcare providers should work together to manage potential oral complications, such as preventing bone loss, mucosal irritation, functional issues, and alveolar ridge resorption. Comprehensive dental rehabilitation and routine recall systems are essential in DM care. Dental implants can be suggested to maintain bone width and height if the disease is controlled [23].

(8) Patients should receive practical measures and recommendations about their oral health in a routine dental care session. The training of diabetes care providers and patients regarding their oral health should be amplified [12].

(9) Retaining natural dentition and periodontal disease treatment and prevention are essential measures for maintaining masticatory performance in older adults [58] and may contribute to the prevention of systemic health status deterioration [17].

(10) DM patients have a special predisposition to the development of fungal infections, especially of the Candida sp. genus, with significantly higher rates of oral mucosa colonization by Candida sp. both in patients with DM1 (85%) and DM2 (68%) compared to non-diabetics (27%) [12]. Change et al. reported that professional dental cleaning was negatively associated with the occurrence of new-onset diabetes [16].

(11) Teeth rehabilitation in edentulous patients is important to reduce embarrassment and increase confidence, which ensures the well-being of the patient. A well-made CD can truly change a person’s life by bringing back a smile on his/her face. Hence, proper postoperative instructions and counseling are vital to keep the patients’ spirits high and motivate them to lead a healthier life.

(12) Replacing missing teeth not only provides adequate nutrition and mastication but also improves physical health. The relationship between physical activity and mortality was compared between partially edentulous (>20 teeth) and edentulous patients (no dentures) on institutionalized senior citizens. Replacing missing teeth in edentulous patients may be critical as they showed a decline in physical ability and an increase in mortality rates [69]. Masticatory rehabilitation should be taken into account as a whole to ensure the holistic treatment of DM patients in particular and to maintain systemic health [34].

(13) Policymakers should improve preventive care approaches and more accurately characterize the care needs of the adult and older adult populations, especially diabetic patients [30].

## Conclusions

Healthcare workers should be aware that patients seeking healthcare, especially older ones, may have dental problems, specifically loss of natural teeth. Loss of teeth may cause these patients to experience malnutrition more than those who have retained their natural teeth. Therefore, during medical or dental visits, all healthcare practitioners should work in a multidisciplinary approach by referring patients to the dentist. The dental examination was suggested according to the literature to focus on the remaining teeth and occlusion status. Early and routine dental care may assist in enhancing oral health and prevent teeth loss and other oral diseases. This is also applied to dietitians; in order to reduce the risk of malnutrition, it is important to recognize tooth loss as part of the oral examination carried out during a nutrition evaluation and by asking questions regarding oral variables affecting one's ability to ingest foods and fluids. In order to improve oral health and terminate additional tooth loss and oral illness, individuals with tooth loss are referred to oral healthcare specialists early on and get inter-professional care. Finally, more long-term studies should be conducted for each community to explore the connection between chewing ability and remaining functional tooth units, using established criteria, clinical factors, methods, and surveys.
